# Advances in Patient Reported Outcomes: Integration and Innovation: Leeds, UK. 13 June 2019

**DOI:** 10.1186/s41687-020-00193-x

**Published:** 2020-05-12

## Advances in Patient Reported Outcomes: Integration and Innovation

### Patricia Holch^1,2^, Kate Absolom^2^, Cat Brooke^1^, Xu Wang^1^

#### ^1^Leeds Beckett University, Centre for Psychological Research, Leeds, United Kingdom; ^2^University of Leeds, Patient Centered Outcomes Research, United Kingdom

Following the success of the previous three PROMs Research Conferences held at University of Sheffield (2016), St Anne’s College, University of Oxford, (2017) and the Centre for Patient Reported Outcomes Research (CPROR) at the University of Birmingham, (2018), we report the proceedings from the 2019 conference held at Leeds Beckett University Centre for Psychological Research (PsyCen) on the 13^th^ June.

**Aims of the conference:**

To gather clinicians, patient partners, researchers, academics, leading international experts, and early career researchers to explore current advances and best practice in research and implementation in the PROM field in the UK and beyond. The overall theme of the conference was **‘Advances in Patient Reported Outcomes:Integration and Innovation’.**

**Summary of event**

Altogether, 77 multi-disciplinary delegates attended including 6 patient representatives. The programme centred around two stimulating plenary sessions, a workshop, parallel oral sessions and poster exhibitions.

**Plenary speakers**

**Mr Roger Wilson, CBE**

***Understanding Value***

Clinical research is evolving fast. New medicine development presents challenges with innovative classes of drug by-passing-controlled studies on the way to approval. Full approval is becoming reliant on ‘real world’ evidence, for which standards and validated methodologies are in their infancy. The concept of VALUE is hard to define in healthcare, unless you are a patient. How to gather and quantify what patients want to say is part of what PROs can provide and offer a route forward which influences regulation, funding/commissioning and clinical care. To do this PROs must evolve from ‘moment in time’ to longitudinal studies and use innovative data gathering techniques which inform disease pathways. To give them full authority patient involvement in their development and analysis is also an imperative, closing the circle on VALUE.

**Founder of Sarcoma UK an active patient representative and advocate with NCRI, NICE, MRC, Cancer Research UK, EORTC, ESMO and EMA.**

**Professor Stephen Radley**

**Web-based PROMs in practice; from Women’s Health to Pre-Operative Assessment**

The initial development of an electronic personal assessment questionnaire was driven by a desire to use validated questionnaires in routine clinical practice; addressing issues of data entry and analysis as well as increasing the value and reducing the burden of questionnaire use (for both patients and clinicians), whilst harnessing the accuracy, reliability and acuity afforded by well-designed PROMs.

Detailed, reliable, objective and meaningful patient self-assessment is now proving valuable, not only for outcomes monitoring, but more importantly, better understanding of patients’ conditions; particularly those of a sensitive or complex nature and when patient’s views of their condition and symptomatology are critically important in informed, shared decision making.

Appropriately designed and deployed web-based self-completed questionnaires can enhance communication and support Virtual Clinics, where elements of patient assessment are conducted remotely and inform consultations and subsequent management. This is proving particularly useful in the context of surgical follow-up, where questionnaires may be combined with scheduled telephone consultation, usually 3-months post operatively. The technology is now being applied more widely in other areas, such as vascular disorders and pre-operative assessment, enabling enhanced communication, efficiency and quality of healthcare. Demonstrable benefits include cost, capacity, patient flow and patient experience.

**Declaration of interest**

Stephen Radley is an unsalaried director and shareholder in ePAQ Systems Ltd, an NHS spin-out technology company, the majority shareholder in which is Sheffield Teaching Hospitals NHS Foundation Trust.

**Consultant Gynaecologist & Director of Research, Sheffield Teaching Hospitals NHS Foundation Trust**

**Workshop**

**‘Improving care with electronic PRO feedback to clinicians: approaches, challenges & solutions’**

**Abstract**

Research has demonstrated that providing individual-level patient-reported outcome (PRO) feedback to clinicians promotes patient self-reflection, improves patient-clinician communication, raises clinicians’ awareness of patient concerns, can be used for remote monitoring and to help patients to share information with their care team. However, there are various challenges to implementing and using these systems, such as development and sustainability of required technology, costs and legal concerns around the status of the electronic system in the context of patient care and ensuring patient and clinician engagement. The purpose of this workshop is to share experiences of designing and/or use of ePROMs feedback systems, challenges encountered and potential solutions, firstly though sharing some results from a systematic review, and secondly through individual case studies. Guided discussion was given for delegates to share their experiences, challenges & solutions of developing similar projects and/or using electronic PRO systems.

**Chair: Galina Velikova, Professor of Psychosocial Oncology, Medical Oncologist, University of Leeds Patient Centred Outcome Research Group and Leeds Teaching Hospitals NHS Trust.**

**Jose M Valderas, Professor of Health Services & Policy Research at the University of Exeter Medical School and a General Practitioner.**

**Kerry Avery, Mairead Murphy, Carmen Tsang & Holly Richards from the University of Bristol**

**Panel discussion**

To close the conference, a panel discussed current ‘Hot topics in PROMS’ chaired by Jennifer Bostock (Patient representative University of Oxford).

The panel discussed the challenges of collecting PROMs from populations with severe cognitive impairment (frail older adults, learning disabilities, Alzheimer’s), how PROM results can be made more readily actionable for clinicians and changing attitudes of the "value added" from PROM data (either at an individual or aggregate/organizational levels). In addition, it was explored how researchers can more actively contribute to i) selecting the most relevant PROMs for each patient group? ii) overcoming implementation problems and foster the clinically relevant interpretation of PROM/PRO data. Finally, they discussed the role of PROMS in integrating care across health and social care services.

The panel members included Galina Velikova (University of Leeds), Georgina Jones, (Leeds Beckett University), Stephen Radley, (Sheffield Teaching Hospitals) Roger Wilson (Patient Representative) and Esther Kwong, (London School of Hygiene & Tropical Medicine)

**Abstracts**

There were 72 abstract submissions and following peer review, 29 were given an oral presentation, 7 were rapid reports and 36 were awarded posters.

Oral presentations

Several sessions were devoted to the conference theme and focussed on *PROM/PRO integration in clinical practice*. Here the presenters showcased the value of patient reported data to support decision making, improve cancer care and achieve value-based health care.

*ePROMS and cutting-edge development*

These talks covered, attitudes of care providers and patients towards ePROs to the support care of patients with traumatic brain injury, developing an integrated national PROMs and PREMs platform for NHS Wales, a taxonomy of short generic person-reported measures and evaluating the feasibility of ePROMS at the Royal Marsden Hospital

*National and International PROM development*

Presentations addressed how the utilisation of PROM/PRO assessments could maximise impact for patients and society including through the NHS Wales large scale PROMs and PREMs initiatives. An international collaboration to develop an immunoglobulin burden of treatment questionnaire for patients with primary immunodeficiencies: the IgBoT-35 and Developing International Standards and Recommendations for the analysis of PROM/PRO data were also covered.

*Health economics*

The impact of having a patient reported outcome measure as a co-primary endpoint on the design, management and analysis of a large phase II/III randomised controlled trial.

*Innovative Patient and Public involvement (PPI)*

Presentations included the role of PPI when implementing PROMs in routine practice and the Unspoken Voices Project describing the co-design of a conceptual framework with people who have complex communication needs and working creatively disseminate a national PROMS study.

*Reporting standards and PROM development*

These talks included development of new PROMS for Polymyalgia Rheumatica and Sarcoma Assessment and using Fourier analysis to examine variations in outcome scores for Meniere’s Disease. The variation in scores across different time-points (day, week, month) for patients with multiple conditions was also explored.

*Diversity, drivers and pilot studies*

These talks included ethnic group recruitment and utilization of appropriate PROMS in cancer clinical trials, a Systematic Review of Self-reported QOL measures for adults with mild to moderate learning disabilities and the importance of pre-testing QOL items for primary sclerosing cholangitis and what influences patient’s measured outcomes.

**Rapid Reports**

Further oral presentations as rapid reports were presented which included an exploration of the performance of the ICECAP-O in cataract surgery patients, web-based surveys for the administration of PROMS and a Delphi consultation informing the development of a community-based follow-up intervention for ovarian cancer patients. Other talks included, symptom burden after living donor kidney transplant, optimising PPI Involvement in PROM development, development of a PREM for the bladder cancer treatment pathway and lessons from a pilot study assessing self-completion and proxy measurements for QOL in children with behavioural problems.

**Prize Winners**

Several prizes were judged and awarded:

**Best Oral: Early Career research award**: **Katherine Broomfield**, ‘The Unspoken Voices Project: Co-designing a conceptual framework with people who have complex communication needs who rely on augmentative and alternative communication’

**Best Oral Rapid Report: Leanne Shearsmith,** ‘electronic Patient self-Reported outcomes to Improve cancer Management and patient Experiences (ePRIME) – a Delphi consultation informing the development of a community-based follow-up intervention for ovarian cancer patients’

**Best PhD Poster: Katharina Vogt,** ‘Development and evaluation of a novel method to validate Clinical Outcome Assessments: the use of vignettes in rare disease research and assessment’

**Oral or Poster**: **Best patient and public involvement award** for the team which have demonstrated excellence and/or innovation in involving the public: **Rachel Taylor, Brian Lobel, Keisha Thompson, Jeremy Whelan, Lorna Fern** ‘Working with young people to creatively disseminate a national PROMS study – developing 'There is a Light: BRIGHTLIGHT’

**Acknowledgements**

Key funding and support was provided by the following NIHR Collaborations for Leadership in Applied Health Research and Care (CLAHRC): Oxford, Yorkshire & Humber, West and East of England. Further support was given from Springer Open and the Journal of Patient Reported Outcomes and the conference was endorsed by the International Society for Quality of Life Research (ISOQOL).

The conferenced gained the ‘Patients Included’ charter mark in recognition of the patient and public involvement in planning, attending, presenting and chairing sessions at the conference.

## Oral sessions

### 1) AMBUFLEX - Implementation of a generic web-based system to support flexible outpatient follow-up by use of PRO for clinical decision support

#### Birgith Engelst Grove^1^, Liv Marit Valen Schougaard^1^, Caroline Trillingsgaard Mejdahl^1^, Louise Pape Larsen^1^, Niels Henrik Hjollund^1,2^

##### ^1^AmbuFlex, Regional Hospital West Jutland, Herning, Denmark; ^2^Department of Clinical Epidemiology, Aarhus University Hospital, Aarhus, Denmark

###### **Correspondence:** Birgith Engelst Grove

**Abstract**

**Background & Aims**

Follow-up visits for patients with chronic conditions in secondary care are traditionally based on pre-booked scheduled appointments. AmbuFlex is a generic web-based system which supports the use of Patient-Reported Outcome (PRO) as the basis for follow-up. PRO collected at home is used to evaluate the needs and wishes for clinical attention. Scheduled appointments are substituted with diagnosis specific questionnaires.

**Method**

The AmbuFlex PRO solutions are customized to each clinical setting, thereby making it possible to manage a diversity of diagnostic groups and clinical work flows. AmbuFlex consists of 3 elements: PRO data collection, a PRO-based automated decision algorithm and a PRO-based graphical overview. We describe our experiences with large-scale implementations of PRO as the basis for follow-up in patients with different chronic and malignant diseases using the generic PRO system AmbuFlex.

**Results**

The overall aim for AmbuFlex is to a) Improve quality of care, b) Support Patient involvement, c) Increase resource reallocation and d) Provide PRO data for clinical research and quality improvement. AmbuFlex has been well integrated into clinical practice in outpatient clinics throughout Denmark since 2011. By March 2019, a total of 25 different patient groups are using PRO with an algorithm to support clinical decision-making. In total 28.256 patients, mean age 55.0 (16.2) have been referred in 65 different departments.

**Conclusion**

The results indicate that it is possible to successfully implement one generic PRO system in a variety of patient groups with chronic and malignant diseases. If knowledge of the patient´s health status can be provided, and only patients with clinical need are seen in the outpatient clinic, resources can be transferred to patients with actual need or wish for a contact. Algorithms used in the treatment of patients with chronic condition is widely utilised and help support decision-making in clinical practice.

### 2) Colorectal cancer survivors’ long-term health-related quality of life (HRQOL): A systematic review of the qualitative evidence

#### Müller Fabiola, Kate White, Nasiba Sadaf Faiz, Madeleine King.

##### Claudia Rutherford University of Sydney, Sydney, Australia

###### **Correspondence:** Müller Fabiola

**Abstract**

**Background & Aims**

Colorectal cancer (CRC) is among the most prevalent cancers in both men and women. Favourable survival rates highlight the need to better understand survivors’ experiences of the long-term impact of CRC and its treatment, which can in turn foster the screening for key patient-reported outcomes (PROs) in clinical practice. The aim of this systematic review was to identify and synthesise CRC survivors’ experiences of a comprehensive range of long-term impacts on HRQOL.

**Method**

We searched Medline, Embase and PsychINFO from inception to January 2019. Qualitative studies describing CRC survivors’ experiences at least 1-year after treatment completion were included. Eligibility and quality assessment, according to COREQ guidelines, was performed independently by two reviewers. Data synthesis was performed by three reviewers and discussed with the study team.

**Results**

Of 1363 papers retrieved, 17 papers reporting 12 studies met eligibility criteria. Seven papers investigated experiences of survivors with a permanent stoma and three with a reversed stoma. Thematic synthesis produced nine themes: symptoms, physical, social, psychological and sexual functioning, impact on relationships, supportive care needs, health care experiences, health behaviour, financial toxicity and occupational experiences. Stoma problems (e.g. leakage, skin irritation) were common in ostomates. Survivors with a reversed stoma experienced unexpected, long-term altered bowel functioning (e.g. incontinence, diarrhoea). Survivors often adjusted their diet (e.g. avoid specific foods) to manage their bowel symptoms. Less commonly reported symptoms include fatigue, impaired sleep and anal pain. Stoma problems and altered bowel functioning impaired survivors’ physical, social, sexual and psychological functioning (e.g. embarrassment). Cognitive functioning and heredity issues were not reported in any paper.

**Conclusion**

CRC survivors experience ongoing impairments more than 1-year after treatment completion. Survivors with a permanent or reversed stoma experience ongoing and impairing bowel symptoms. Follow-up healthcare should integrate screening for likely long-term PROs and provide targeted supportive care.

### 3) The use of electronic Patient Reported Outcome Measures (ePROMs) to revolutionise cancer care

#### Marianna Christodoulou^1^, Nada Khalil^1^, Philip Rust^1^, David Thomson^1^, Ed Smith^1^, Sacha Howell^1^, James Price^1^, Jacqueline Fenemore^1^, Hilary Neal^1^, Marie Eaton^1^, Matthew Barker-Hewitt^1^, Liam Orrell^1^, Jo Jackson^1^, Wes Dale^1^, Robert Bristow^1,2,3^, Corinne Faivre-Finn^1,2^, Janelle Yorke^1,4^

##### ^1^The Christie NHS Foundation Trust, Manchester, United Kingdom; ^2^Division of Cancer Sciences, University of Manchester, Manchester, United Kingdom; ^3^Manchester Cancer Research Centre, Manchester, United Kingdom; ^4^Division of Nursing, Midwifery & Social Work, University of Manchester, Manchester, United Kingdom

###### **Correspondence:** Marianna Christodoulou

**Abstract**

**Background & Aims**

Collection of Patient Reported Outcome Measures (PROMs) in oncology has been associated with improved patient outcomes in the context of clinical trials. The Christie NHS Foundation Trust is, to our knowledge, the first cancer centre worldwide to introduce electronic PROMs (ePROMs) as a standard service in clinical practice.

**Methods**

The Christie ePROMs group worked with clinical teams and patient groups to adapt the Common Terminology Criteria for Adverse Events (v4.03) into symptom ePROM tools. Patients complete symptom-based ePROMs alongside a Quality of Life (QoL) tool (EQ-5D-5L). Patients receive advice on the management of their symptoms based on their responses. An ePROMS platform, ‘MyChristie-MyHealth’ was developed. The service has received local Information Governance Caldicott approval and clinical safety sign-off. Phase 1 of the initiative involves roll out of MyChristie-MyHealth to all patients with cancers of the lung or head and neck and to those treated with Proton Beam Therapy. Phase 2 includes roll out to the rest of the Trust by 2020.

**Results**

From January 2019, MyChristie-MyHealth has been available to a proportion of lung and head and neck cancer patients as part of phase 1 of the initiative. Patients receive a text message or email with a web link 3 days prior to their outpatient clinic appointment. The link allows patients to access and complete ePROMs remotely in between their clinic appointments. Clinical teams are able to review their patients’ responses through an online clinical portal. As of week, 3 of phase 1, we have achieved a completion rate of 41% without initial patient prompting.

**Conclusion**

The introduction of ePROMs as a service in routine clinical practice is feasible. Future work will focus on formal evaluation of the impact of MyChristie-MyHealth to patients and the Trust. We are developing patient prompts to further increase completion rates.

### 4) The utility of PROMS in measuring the value of care pathways

#### Lucinda Gabriel, Joseph Casey

##### King’s Health Partners, London, United Kingdom

###### **Correspondence:** Lucinda Gabriel

**Background**

In 2009, NHS England introduced the routine measurement of patient reported outcomes measures (PROMS) with a view to putting health care outcomes at the centre of NHS decision-making. In 2012 the program stalled however, and only four nationally mandated programs continue to exist. With renewed interest in creating a value-driven NHS as outlined in the ‘Five Year Forward View’ sharing outcomes that matter to patients and the cost of care pathways is key

**Aims**

Our aim was to evaluate how PROMS could be used to inform the value of a surgical intervention in terms of the quality of care provided to patients, and the efficiency with which that care is delivered. Two distinct surgical care pathways for the treatment of primary hip osteoarthritis were evaluated to determine which elements may better promote the delivery of high-value clinical care.

**Methods**

Two care models were evaluated: a traditional model with multiple entry points and without pathway standardisation, and an intentionally designed standardised multidisciplinary pathway. NHS mandated PROMS (Oxford Hip Score, EQ-5D, EQ- VAS) were extracted from national databases. These measures were then restructured into a patient-centred format to assess the impact on pain, function and psychological outcomes. The intention being this format would resonate more meaningfully with patients. A patient level economic costing exercise was undertaken in an effort to develop clinically meaningful cost information to inform pathway redesign.

**Results**

Clinical outcomes showed improvement in all domains with little variation across the two models. Individual scores did not show uniform improvement. The intentionally designed model delivered better value care, demonstrating a small positive financial margin.

**Conclusions**

Analysis of the two care pathways showed an intentionally designed pathway delivers comparable outcomes at lower cost. Developing and measuring patient-focussed outcomes will inform rational economic evaluation of the health service and are key to understanding and achieving better value care. An evaluation of how PROMs can be communicated to patients more meaningfully to inform care decisions should follow.

^1^NHS England, Care Quality Commission, Health Education England, Monitor, Public Health England, Trust Development Authority (2014). NHS *five-year forward view*. London: NHS England. Available at: www.england.nhs.uk/ourwork/futurenhs/ (accessed on 2 February 2019).

### 5) Maximising the impact of patient reported outcome assessment for patients and society

#### Melanie Calvert^1^, Derek Kyte^1^, Gary Price^2^, Jose M Valderas^3^, Niels Henrik Hjøllund^4^

##### ^1^Centre for Patient Reported Outcomes Research (CPROR), Institute of Applied Health Research, and NIHR, Birmingham Biomedical Research Centre, University of Birmingham, Birmingham, United Kingdom; ^2^Centre for Patient Reported Outcomes Research (CPROR), University of Birmingham, Birmingham, United Kingdom; ^3^NIHR PenCLAHRC and Institute for Health Services Research, University of Exeter Medical School, St Luke’s Campus, St Leonard’s, Exeter, United Kingdom; ^4^AmbuFlex/WestChronic, Regional Hospital West Jutland, Herning, Denmark, and Department of Clinical Epidemiology, Aarhus University Hospital, Aarhus, Denmark

###### **Correspondence:** Melanie Calvert

**Abstract**

**Background & Aims**

Patient reported outcome (PRO) data are increasingly used by a range of stakeholders to support communication between patients and healthcare professionals, inform health technology assessment, pharmaceutical labeling claims, health policy and service improvement. These data may offer major benefits to patients and society, but current use is fragmented and sub-optimal.

**Methods**

We will present the results of our recent BMJ analysis piece (BMJ 2019;364: k5267) providing an overview of the current use and benefits of PROs, challenges with PRO assessment and key considerations for a more integrated approach to PRO assessment.

**Results**

Key challenges with current PRO assessment include: the selection of appropriate measures; ethical issues such as patient burden and management of PRO alerts; optimal data collection, analysis, reporting, and interpretation; and data logistic issues including integration with the electronic health record and lack of coordination within and across clinical specialties/healthcare systems to meet multiple stakeholder needs. Key considerations for integrated PRO assessment include: stakeholder engagement and cooperation; coordinated efforts to identify and select appropriate outcomes; ensuring appropriate governance; development of health informatics infrastructure; and further developments in methods for the analysis, interpretation and dissemination of PRO data. Iterative improvements of PRO system development, assessment of the impact of PRO systems on clinical workflow and patient outcomes, and establishing the cost-effectiveness of PRO systems will be essential.

**Conclusion**

Routine collection, processing, and sharing of PRO data may offer huge benefits to patients and society. A crucial first step is to establish a national multi-stakeholder steering group, involving patients, clinicians, PRO methodologists, regulators, policy makers and NHS digital, aimed at standardizing PRO data capture and consolidating/sharing knowledge and good practice.

### 6) NHS Wales large scale PROMs and PREMs collection to drive improvement and to achieve a value-based healthcare transformation

#### Sarah Puntoni

##### Cardiff and Vale University Health Board, Cardiff, United Kingdom

**Abstract**

**Background & Aims**

The all Wales PROMs, PREMs and Effectiveness Programme aims to collect PROMs (Patient Reported Outcome Measures) and PREMs (Patient Reported Experience Measures) across secondary care. Its remit includes the development of an electronic portal to capture the data, integrated into the existing National informatics architecture.

Since being launched in January 2016, PROMs ‘pathways’ for 30 clinical conditions have been nationally agreed, built and are can be collected via the national PROMs/PREMs portal. Alongside each condition specific PROM, a generic set (which includes EQ5D) is also collected to allow population level analysis and comparison across specialties/ pathways.

**Methods**

The Programme is fully bilingual and the national team is responsible for negotiating licenses, and where required, undertaking Welsh language translation and validation according to license holder requirements and following ISPOR guidance.

PROMs collection is currently live across Wales and to date over 25,000 PROMs have been collected, most of which are at point of referral into secondary care. Longitudinal collection is expanding, with pockets of collection at post-treatment and follow up stages.

The Programme also led the validation of a universal PREM survey, now part of the Welsh Government Framework for measuring service user experience; the first PREM pilot was launched in January 2019.

The data collected is immediately available into the patient’s electronic record and PROM data visualization tools are being piloted in lung cancer to aid shared decision making. Health Boards access their data weekly.

**Conclusion**

All data collected is also held within the National Data Repository, allowing data to be linked to other nationally held datasets for advanced analytics. A pilot study, linking Orthopaedic PROMs to OPCS clinical coding, National Joint Registry and costing data is underway.

The team will share their experience and lessons learnt, including examples of early analysis.

### 7) An international collaboration to develop an immunoglobulin burden of treatment questionnaire for patients with primary immunodeficiencies: the IgBoT-35

#### Georgina Jones^1^, Mark Edmondson-Jones^2^, Leire Solis^3^, Johan Prevot^3^, Jose Drabwell^3^, Anna Shrimpton^4^, Nizar Mahlaoui^5^

##### ^1^Leeds Beckett University, Leeds, United Kingdom; ^2^Parexel, London, United Kingdom; ^3^IPOPI, Cornwall, United Kingdom; ^4^Sheffield Teaching Hospitals, Sheffield, United Kingdom; ^5^Necker -Enfants Malades University Hospital, Paris, France

###### **Correspondence:** Georgina Jones

**Abstract**

**Background & Aims**

Burden of treatment describes the effort of being a patient. We describe the international development and psychometric testing of a new questionnaire to measure the burden of immunoglobulin treatment as reported by patients with primary immunodeficiencies (the IgBoT-35).

**Methods**

The IgBoT-35 was developed in collaboration with an international team involving academic, industry and patient partners. In four stages, we undertook: i) evidence synthesis and appraisal of the existing literature, ii) open-ended exploratory interviews with 30 adult patients (aged 16 years and over), iii) a face validity exercise involving an additional 14 patients, and an iv) online, cross-sectional survey across 10 countries (nine European and Canada) to reduce the measure and identify its reliability, domain structure and scoring algorithms.The questionnaire was translated following the guidance of the ISPOR task force.

**Results**

Patients were invited to participate in the study by the patient partners and local national member organisations (NMOs). In total, 472 patients completed the online questionnaire, of which 395 were included in the study (32% underwent intravenous Ig treatment and 67% underwent subcutaneous Ig Treatment). The final instrument contained eight domains: Time (4 items), Organisation and Planning (5 items), Leisure (5 items), Interpersonal Relationship (3 items), Employment and Education (3 items), Travel (5 items), Consequences of Treatment (6 items), and Emotional (3 items). An additional Ig global treatment burden question was included at the end of the measure (n=35 items).

**Conclusion**

The Ig-BoT-35 appears to be a reliable, patient-generated questionnaire. A further survey has recently been undertaken in a new sample of US patients to further establish the validity and test the conceptual model of the measure. When used in clinical practice it may help to better understand reasoning behind treatment choice and thus identify the decision support needs of patients with primary immunodeficiencies facing Ig treatment choices.

### 8) Developing International Standards and Recommendations for the Analysis of Patient-Reported Outcomes and Quality of Life Data

#### Lien Dorme^1^, Madeline Pe^1^, Corneel Coens^1^, Ethan Basch^2^, Melanie Calvert^3^, Alicyn Campbell^4^, Charles Cleeland^5^, Kim Cocks^6^, Laurence Collette^1^, Nancy Devlin^7^, Amylou C Dueck^8^, Hans-Henning Flechtner^9^, Carolyn Gotay^10^, Ingolf Griebsch^11^, Mogens Groenvold^12^, Laura Lee Johnson^13^, Madeleine King^14^, Paul G Kluetz^13^, Michael Koller^15^, Daniel C Malone^16^, Fancesca Martinelli^1^, Sandra A Mitchell^17^, Jammbe Z Musoro^1^, Daniel O'Connor^18^, Kathy Oliver^19^, Elisabeth Piault-Louis^4^, Martine Piccart^20^, Chantal Quinten^21^, Jaap C Reijneveld^22^, Christoph Schürmann^23^, Jeff Sloan^24^, Ashley Wilder Smith^17^, Katherine M Soltys^25^, Rajeshwari Sridhara^13^, Martin J B Taphoorn^26,27^, Galina Velikova^28^, Andrew Bottomley^1^, *Written On behalf of the Setting International Standards In Analyzing Patient-Reported Outcomes and Quality of Life Endpoints Data (SISAQOL) Consortium’*

##### ^1^EORTC, Brussels, Belgium; ^2^Lineberger Comprehensive Cancer Center, Chapel Hill, USA; ^3^Centre for Patient Reported Outcomes Research, Institute of Applied Health Research, College of Medical and Dental Sciences, University of Birmingham, Birmingham, United Kingdom; ^4^Genentech, San Francisco, USA; ^5^University of Texas MD Anderson Cancer Center, Houston, USA; ^6^Adelphi Values, Bollington, United Kingdom; ^7^University of Melbourne, Melbourne, Australia; ^8^Alliance Statistics and Data Center, Mayo Clinic, Scottsdale, USA; ^9^Clinic for Child and Adolescent Psychiatry and Psychotherapy, Universität Magdeburg, Magdeburg, Germany; ^10^School of Population and Public Health, University of British Columbia, Vancouver, Canada; ^11^Boehringer Ingelheim International GmbH, Ingelheim, Germany; ^12^Department of Public Health; Bispebjerg Hospital and University of Copenhagen, Copenhagen, Denmark; ^13^Office of Hematology and Oncology Products, Center for Drug Evaluation and Research, US Food and Drug Administration (FDA), Silver Spring, USA; ^14^School of Psychology and Sydney Medical School, University of Sydney, Sydney, Australia; ^15^Center for Clinical Studies, University Hospital Regensburg, Regensburg, Germany. ^16^College of Pharmacy, University of Arizona, Tucson, USA; ^17^National Cancer Institute (NCI), Bethesda, USA; ^18^Medicines and Healthcare products Regulatory Agency (MHRA), London, United Kingdom; ^19^International Brain Tumour Alliance (IBTA), Surrey, United Kingdom; ^20^Institut Jules bordet, Université Libre de Bruxelles, Brussels, Belgium; ^21^European Centre for Disease Prevention and Control (ECDC), Surveillance and Response Support Unit, Epidemiological Methods Section, Stockholm, Sweden; ^22^VU University Medical Center, Department of Neurology & Brain Tumor Center, Amsterdam, Netherlands; ^23^Institute for Quality and Efficiency in Health Care (IQWIG), Cologne, Germany; ^24^Alliance Statistics and Data Center, Mayo Clinic, Rochester, USA; ^25^Health Canada, Ottawa, Canada; ^26^Leiden University Medical Center, Leiden, Netherlands; ^27^Medical Center Haaglanden, The Hague, Netherlands; ^28^Leeds Institute of Cancer and Pathology, University of Leeds, St James's Hospital, Leeds, United Kingdom

###### **Correspondence:** Lien Dorme

**Abstract**

**Background & Aims**

Patient-reported outcome (PRO) data, such as health-related quality of life and symptoms, are increasingly being captured in cancer randomized clinical trials (RCTs) to provide valuable information on treatment risks, benefits and tolerability. Our literature review in various cancer fields showed little consensus about the analysis, interpretation and reporting of these data, hindering comparability of and confidence in results across trials. The Setting International Standards in Analyzing Patient-Reported Outcomes and Quality of Life Endpoints Data (SISAQOL) Consortium was convened to set recommendations for PRO analysis in cancer RCTs.

**Methods**

The Consortium is composed of 40 international experts including PRO researchers and statisticians, representatives from regulatory bodies, academic societies, pharmaceutical industry, cancer institutes and patient organizations. Subgroups were formed to focus on four priorities: (a) specification of well-defined PRO research objectives, (b) recommendations for appropriate statistical PRO analysis methods, (c) standardization of statistical terminology and (d) development of guidelines for analyzing missing data. Methods used included literature review, surveys, and expert discussions in teleconferences and face-to-face meetings. Recommendations were ratified through consensus voting in a final meeting.

**Results**

A taxonomy of research objectives was established. Appropriate statistical methods, with the exception of summary measures, were proposed. Consensus was reached on the taxonomy of research objectives and statistical methods, along with a definition of missing data and two rates to report missing data occurrence. While some statements concerning handling missing data or statistical analyses are still to be discussed, many statements were ratified for each of the priorities.

**Conclusion**

A robust first set of PRO analyses recommendations was developed in a joint process with diverse international stakeholders. Addressing the needs and requirements of these stakeholders provides a strong foundation for widespread endorsement of these recommendations. Ultimately, harmonization of current research practices will enhance interpretability and impact of PRO data in cancer RCTs.

*Disclaimer*

*The views here reflect that of the individual authors and should not be construed to represent official views or policies of the US Food and Drug Administration, US National Cancer Institute, Medicines and Healthcare products Regulatory Agency, Institute for Quality and Efficiency in Health Care, Germany or Health Canada.*

### 9) The impact of having a patient reported outcome measure as a co-primary endpoint on the design, management and analysis of a large phase II/III randomised controlled trial

#### Kara-Louise Royle^1^, Janet Brown^2^, Fiona Collinson^1^, Christy Ralph^3,4^, Jayne Swain^1^, Rebecca Day^1^, Patrick Hanlon^5^, Adam Martin^6^, David Meads^6^, Walter Gregory^1^, Lucy McParland^1^

##### ^1^Clinical Trials Research Unit, Leeds Institute of Clinical Trials, University of Leeds, Leeds, United Kingdom; ^2^University of Sheffield, Sheffield, United Kingdom; ^3^University of Leeds, Leeds, United Kingdom; ^4^Leeds Teaching Hospitals, Leeds, United Kingdom; ^5^Patient Representative National Cancer Research Institute (NCRI) Renal Cancer Clinical Studies Group, Leeds, United Kingdom; ^6^Academic Unit of Health Economics, Leeds Institute of Health Sciences, University of Leeds, Leeds, United Kingdom

###### **Correspondence:** Kara-Louise Royle

**Abstract**

**Background & Aims**

STAR is a phase II/III, UK multicenter, randomised controlled, non-inferiority trial evaluating the use of treatment breaks for patients with renal cancer compared with continuous treatment.

There is evidence that systemic therapy treatment breaks may be associated with reduced toxicity and improved Quality of life (QoL), without compromising long-term efficacy. Therefore, patient reported outcome measures (PROMs) were a key motivator for the trial. Consequently, quality adjusted life-years (QALYs), calculated using the patient reported EQ-5D questionnaire up to a minimum of 2-years follow-up, were included as a co-primary endpoint.

We will report on the impact this innovative decision has had on the design, management and planned analysis of the STAR trial.

**Methods**

At trial design there was limited evidence available to calculate the power for the QALYs endpoint. Simulations were conducted under several assumptions, which were to be re-assessed at the end of phase II.

During treatment and follow-up, research staff training, reminder text messages to patients and both postal and in-clinic questionnaire completion were some of the methods used to maximise compliance of the EQ-5D questionnaire. Additionally, reasons for missing QoL data were collected and multiple imputation will be used to impute any missing EQ-5D data.

**Results**

Between 2011 and 2017, 920 participants were recruited. The trial is currently in follow-up; therefore, the implemented methods cannot currently be evaluated. One key finding has been that despite reassurance from patient and public representatives, research staff have questioned the appropriateness of sending longer-term follow-up questionnaires to patients, particularly those in not on active treatment.

**Conclusion**

The importance of including PROMs in clinical trials is widely acknowledged. However, including a PROM as a primary endpoint requires proactive management at sites and specific consideration when planning the analyses, in order to both minimise the extent of, and appropriately account for, missing data.

### 10) The role of Patient and Public Involvement when implementing PROMs in routine practice

#### Alexis Foster, Alicia O'Cathain, Janet Harris

##### University of Sheffield, Sheffield, United Kingdom

###### **Correspondence:** Alexis Foster

**Abstract**

**Background & Aims**

Increasingly, Patient and Public Involvement (PPI) plays an essential role in the development of Patient Reported Outcome Measures (PROMs). However less understood is the role of PPI in implementing PROMs in routine practice and how approaches may vary between organisations. Purpose: To understand how PPI facilitates the implementation of PROMs in routine practice, using the third sector (for example charities and community groups) as a case study.

**Method**

Thirty interviews were undertaken with a range of third sector stakeholders including service-users, front-line workers, managers and commissioners from across the UK to explore the facilitators and barriers to implementing PROMs. The role of PPI in implementing PROMs was a key issue identified in the analysis.

**Results**

Organisations rarely consulted service-users when implementing PROMs, despite having undertaken PPI when designing front-line services. Three approaches to PPI were identified. First, some organisations did not undertake PPI nor consider their users’ specific needs when implementing PROMs. Sometimes this was because a commissioner had imposed PROMs on the organisation. Consequently, these organisations often had low completion rates of PROMs and had to redesign the original PROMs process. Second, some organisations considered their users’ needs when implementing PROMs, but did this through their workers’ perceptions of these needs rather than consulting users directly. Lastly, some organisations proactively involved users in implementing PROMs such as selecting which PROM to use. These organisations felt that undertaking PPI had been an important facilitator in implementing PROMs successfully. Many of the interviewees discussed how their organisations could have undertaken more PPI in relation to PROMs, indicating that PPI may increase as organisations learn from each other’s successes.

**Conclusion**

Investing time in undertaking PPI is important because it appears to facilitate the sustainable use of PROMs within routine practice.

### 11) Identifying Symptoms Clusters among Pediatric Chronic Kidney Disease Patients Using PROMIS® Computer Adaptive Tests

#### Devin Peipert, Robert Chapman, Michelle Langer, David Cella, Jin-Shei Lai

##### Department of Medical Social Sciences, Northwestern University Feinberg School of Medicine, Chicago, USA

###### **Correspondence:** Devin Peipert

**Abstract**

**Background & Aims**

Identifying symptoms clusters (SCs) helps characterize patients’ health and enhances treatment planning. The Patient Reported Outcomes Measurement Information System (PROMIS®) captures SCs in very brief computer adaptive tests (CATs) based on validated item banks more reliably than standard, one-item-per-symptom assessments.

**Method**

We used data from 384 pediatric chronic kidney disease (CKD) patients (42% female, mean age = 13 years) on PROMIS pediatric mobility (MOB), upper extremity functioning (UE), depressive symptoms (DEP), anxiety (ANX), and fatigue (FAT) CATs. PROMIS CAT scores are reported with a T-score metric (mean = 50, SD = 10), and higher scores indicate more of the measured construct (e.g., higher DEP scores indicate more depression). We modeled SCs at the domain level using two statistical approaches: bifactor exploratory analysis (EFA, oriented toward correlations among symptoms) and latent profile analysis (LPA, oriented towards identifying profiles of patients in which symptoms co-occur). Each PROMIS CAT T-score was entered into each model.

**Results**

Mean CAT T-scores were: MOB = 51.5; UE = 50.1; DEP = 45.7; ANX = 46.3; FAT = 47.3. The bifactor EFA showed that a general factor representing overall symptom burden accounted for most of the variance (67%), suggesting that the PROMIS CATs tapped a similar construct (omega reliability = 0.88). The LPA suggested 3 SCs mapping onto symptom severity: High Burden/Low Function (MOB T-score: 44.4; UE: 44.5; DEP: 58.6; ANX: 58.0; FAT: 53.2), Average Function/No Burden (MOB T-score: 53.7; UE: 51.6; DEP: 43.0; ANX: 42.4; FAT: 49.2), and High Function/No Burden (MOB T-score: 56.0; UE: 54.7; DEP: 36.4; ANX: 36.0; FAT: 30.6).

**Conclusion**

Among pediatric CKD patients, symptoms as measured by PROMIS were clustered, and patients were characterized to different severity-based profiles. Sourcing PROMIS CATs for symptom identification is a reliable, clinically-feasible method for determining whether pediatric CKD patients experience high vs. low symptom burden.

### 12) The Unspoken Voices Project: Co-designing a conceptual framework with people who have complex communication needs who rely on augmentative and alternative communication

#### Katherine Broomfield^1,2^, Karen Sage^1^, Simon Judge^3^, Claire Craig^1^, Georgina Jones^4^

##### ^1^Sheffield Hallam University, Sheffield, United Kingdom; ^2^Gloucestershire Care Services NHS Trust, Gloucester, United Kingdom; ^3^Barnsley Hospital NHS Foundation Trust, Barnsely, United Kingdom; ^4^Leeds Beckett University, Leeds, United Kingdom

###### **Correspondence:** Katherine Broomfield

**Abstract**

**Background & Aims**

This NIHR-funded research study aims to develop a PROM for people who use augmentative and alternative communication (AAC). People with complex communication needs (CCN), usually resulting from a neurological condition, have difficulties with speaking or writing. AAC are tools used by some people with CCN to help them communicate and range from basic, paper-based resources to complex computer systems. Generating meaningful patient and public involvement (PPI) with people who have CCN is challenging; new and innovative approaches are needed. This study provides an overview to date of the methodology developed and used to better facilitate, engage, and support PPI activity during the development of this PROM.

**Method**

A PPI group was formed, consisting of 7 members who use AAC, to support and advise the research team. Participatory design (PD) is an emancipatory co-design approach. PD principles have been used to enable engagement in meetings to help overcome some of the physical and communication barriers to inclusion faced by group members. A number of methods have supported the PPI group, including: 1: Using an artist to graphically minute meetings; 2. Providing audio-visual meeting information and 3. Sorting and rating objects, pictures, words and phrases.

**Results**

To date 5 meetings have been held with the PPI group. The group has successfully informed the development of the conceptual framework for the PROM through: 1. Generating a topic guide for qualitative interviews, 2. Validating the preliminary data synthesis of two systematic reviews. They have also supported the development of easy-read summaries of project outputs.

**Conclusion**

Co-design principles and methods have enabled the inclusion of people who use AAC in meaningful involvement in this PROM development project. The lessons learned and methodologies adopted may also be useful to other developers of PROMS working with patient groups with similar complex and challenging healthcare needs.

### 13) Working with young people to creatively disseminate a national PROMS study – developing *'There is a Light: BRIGHTLIGHT’*

#### Rachel Taylor^1^, Brian Lobel^2^, Keisha Thompson^3^, Jeremy Whelan^1^, Lorna Fern^1^

##### ^1^University College London Hospitals NHS Foundation Trust, London, United Kingdom; ^2^The Royal Central School of Speech and Drama, London, United Kingdom; ^3^Contact Theatre, Manchester, United Kingdom

###### **Correspondence:** Rachel Taylor

**Abstract**

**Background & Aims**

A key challenge in patient-reported outcome research is the translation of results into evidence that informs practice. Social networks are being increasingly noted as influencing how research evidence is integrated into practice and the arts have been proposed as an ideal format as they facilitate subjective interpretation and construction of personal meaning. In this paper we evaluate the impact of working with Contact Young Company (CYC) who developed an hour-long performance based on their interpretation of BRIGHTLIGHT results. BRIGHTLIGHT is the national evaluation of teenage and young adult cancer services in England.

**Method**

We presented BRIGHTLIGHT results during five workshops to 20 members of CYC and four young people (YP) with cancer who contextualised the results. In the subsequent 4-weeks the CYC created the performance. ‘There is a Light’ was performed 11 times in seven UK cities, including international nursing research, international oncology and patient conferences. Data evaluating the impact of the performance includes video diaries from the cast and audience surveys.

**Results**

Over 1300 people attended the performances and >600 viewed a live stream. The cast found cancer in YP a challenging subject to address with the majority having limited/no prior experience of cancer. Young people were supported through this by the producer/director and the YP with cancer. Those with cancer found the experience helped restore their confidence. Feedback from the audience indicated the performance raised awareness and issues around cancer in YP in a meaningful way.

**Conclusion**

Theatre enabled BRIGHTLIGHT results to be viewed by a diverse audience, in greater numbers than traditional methods of dissemination. Although the cast felt this was a challenging subject, they respectfully interpreted BRIGHTLIGHT results and conveyed the results in a meaningful way.

### 14) How easy to read are patient-reported outcome measures (PROMs) used in ophthalmology?

#### Deanna Taylor, Lee Jones, Laura Edwards, David Crabb

##### City, University of London, London, United Kingdom

###### **Correspondence:** Deanna Taylor

**Abstract**

**Background & Aims**

Patient-reported outcome measures (PROMs) are commonly used in clinical trials and research in ophthalmology. Yet in order to be effective, the PROM needs to be understandable to its respondents. The aim of this study was to assess the reading comprehension level of PROMs validated for use in common eye conditions.

**Methods**

Twenty-four PROMs that had been previously validated for use in at least one of three common ophthalmological conditions (age-related macular degeneration, glaucoma and/or diabetic retinopathy) were included in this study. Reading comprehension level determines the readability that a text must have so that a reader understand the written materials; these were calculated using the Flesch-Kincaid Grade Level test, the FORCAST test, and the Gunning-Fog test using readability calculations software package Oleander Readability Studio 2012.1. The American Medical Association (AMA) and the National Institutes of Health (NIH) recommend readability of patient materials should not exceed a sixth-grade reading level. Number of PROMs requiring a reading level exceeding this threshold was calculated.

**Results**

Median (interquartile range; IQR) readability scores were 6.7 (5.0, 9.3), 9.45 (8.6, 10.1) and 7.5 (5.7, 8.4) for the Flesch-Kincaid Grade Level test, the FORCAST test, and the Gunning-Fog test respectively. Depending on the metric used this meant 58% (95% confidence interval [95% CI] 37 to 78%), 100% (95% CI 85 to 100%) and 71% (49 to 87%) fell outside the 6th Grade reading level recommended by the AMA and NIH.

**Conclusion**

Over one half of the PROM questionnaires and instruments commonly used in ophthalmology require a reading comprehension level better than that recommended by the AMA and NIH for patient material. Some PROMs likely contain questions that are at a level too advanced for most patients to comprehend. Greater care is needed in designing PROMs appropriate for the literacy level of a population.

### 15) Are patient self-reported outcome measures (PROMs) sensitive enough to be used as endpoints in clinical trials? *Evidence from the United Kingdom Glaucoma Treatment Study*

#### Lee Jones^1^, David Garway-Heath^2^, Augusto Azuara-Blanco^3^, David Crabb^1^

##### ^1^City, University of London, London, United Kingdom; ^2^National Institute for Health Research Biomedical Research Centre at Moorfields Eye Hospital, London, United Kingdom; ^3^Queen’s University Belfast, Belfast, United Kingdom

###### **Correspondence:** Lee Jones

**Abstract**

**Background & Aims**

The UK Glaucoma Treatment Study (UKGTS) demonstrated the effectiveness of treatment in patients with glaucoma. We test the hypothesis that responses on PROMs differ between patients receiving a topical prostaglandin analogue (Latanoprost) or placebo eye drops.

**Methods**

Newly diagnosed glaucoma patients recruited into the UKGTS with baseline and exit PROM data (n= 182 and n=168 patients from the treatment and placebo group, respectively). The UKGTS was a multi-centre, randomised, triple-masked, placebo-controlled trial, where patients with newly diagnosed glaucoma were allocated to receive Latanoprost (treatment) or placebo; the observation period was 24-months. Patients completed general health PROMs (EQ-5D and SF-36) and PROMs specific to glaucoma (GQL-15 and GAL-9) at baseline and at exit from the trial. Percentage change between baseline and exit measurement on PROMs were calculated for each patient and compared between treatment arms. In addition, differences between stable patients (n=272) and those with glaucomatous progression (n=78) were assessed.

**Results**

Average percentage change on PROMs was similar for patients in both arms of the trial with no statistically significant differences between treatment and placebo groups (EQ-5D, p = 0.98; EQ-5D VAS, p = 0.88; SF-36, p = 0.94, GQL-15, p = 0.66; GAL-9, p = 0.87). There were statistically significant differences between stable and progressing patients on glaucoma-specific PROMs (GQL-15, p = 0.02; GAL-9, p = 0.02) but not on general health PROMs (EQ-5D, p = 0.62; EQ-5D VAS, p = 0.23; SF-36, p = 0.65)

**Conclusion**

Average change in PROMs on health-related and vision-related quality of life was similar for the treatment and placebo groups. PROMs, may not be sensitive enough to be used as a primary endpoint in clinical trials when participants have newly diagnosed early stage glaucoma.

### 16) Are PROMs just for patients? Piloting the Long-Term Conditions Questionnaire for use with patients and carers in memory clinic settings

#### Caroline Potter^1^, Michele Peters^1^, Maureen Cundell^2^, Rupert McShane^2^, Ray Fitzpatrick^1^

##### ^1^University of Oxford, Oxford, United Kingdom; ^2^Oxford Health NHS Foundation Trust, Oxford, United Kingdom

###### **Correspondence:** Caroline Potter

**Abstract**

**Background & Aims**

The Long-Term Conditions Questionnaire (LTCQ) was developed for assessing the overall impact of long-term health conditions (LTCs) on quality of life. Enhancing quality of life for people affected by dementia is a key focus of English health policy. Acknowledging that most dementia patients are supported by an informal carer, and noting LTCQ’s general construct of ‘living well’ whilst managing illness, we tested LTCQ’s potential for use with both patients and carers in memory clinic settings.

**Methods**

Participants were recruited through one of 14 memory clinics in South East England. Surveys including the LTCQ/LTCQ-Carer, EQ5D (5-level version), and ASCOT-Carer (carer surveys only) were distributed by memory clinic staff from February-September 2018 and returned by post.

**Results**

Patients (n=105) had a mean age of 79 years (range 58-91), with multi-morbidity reported for 78% of the sample. Carers (n=107) had a mean age of 67 years (range 41-90), with 57% reporting a long-term health condition. For both measures, missing data was low (5% or less per item), internal consistency was high (Cronbach’s α=0.93 and α=0.95 for LTCQ and LTCQ-Carer, respectively), and all items correlated with a single general construct. Scores for LTCQ (mean 71.0 of max 100, SD=18.9) and LTCQ-Carer (mean 72.4 of max 100, SD=19.3) correlated moderately with EQ5D scores with less skew towards the most positive health state. LTCQ-Carer scores correlated strongly with ASCOT-Carer scores while covering a broader range of content. Memory clinic staff reported that offering LTCQ-Carer during assessment visits encouraged early dialogue about carer needs.

**Conclusions**

Patients with mild/moderate memory problems, many of whom have multiple LTCs, can complete LTCQ as a meaningful measure of ‘living well’ following diagnosis of MCI or dementia. LTCQ-Carer shows promise as a concurrent measure for evaluating how well carers are supported during the post-diagnosis period.

### 17) Systematic evaluation of patient-reported outcome (PRO) protocol content and reporting in cancer clinical trials (EPiC): qualitative study findings

#### Ameeta Retzer^1^, Melanie Calvert^1,2^, Khaled Ahmed^1^, Thomas Keeley^3^, Jo Armes^4,5^, Julia M Brown^6^, Lynn Calman^4,7^, Anna Gavin^4,8^, Adam Glaser^4,9^, Diana M Greenfield^4,10^, Anne Lanceley^4,11^, Rachel M Taylor^4,12^, Galina Velikova^9^, Michael Brundage^13^, Fabio Efficace^14^, Rebecca Mercieca-Bebber^15,16^, Madeleine T King^15^, Derek Kyte^1,2,4^

##### ^1^Centre for Patient-Reported Outcomes Research, Institute of Applied Health Research, University of Birmingham, Birmingham, United Kingdom; ^2^NIHR Birmingham Biomedical Research Centre, University Hospitals Birmingham NHS Foundation Trust and University of Birmingham, Birmingham, United Kingdom; ^3^GlaxoSmithKline (formerly of CPROR, University of Birmingham), London, United Kingdom; ^4^UK National Cancer Research Institute (NCRI) Psychosocial Oncology and Survivorship CSG subgroup: Understanding and measuring the consequences of cancer and its treatment, London, United Kingdom; ^5^School of Health Sciences, University of Surrey, Guildford, United Kingdom; ^6^Clinical Trials Research Unit, University of Leeds, Leeds, United Kingdom; ^7^Macmillan Survivorship Research Group, Health Sciences, University of Southampton, Highfield Campus, Southampton, United Kingdom; ^8^N. Ireland Cancer Registry, Centre for Public Health, Queens University, Belfast, United Kingdom; ^9^Leeds Institute of Medical Research at St James’s, University of Leeds, Leeds, United Kingdom; ^10^Sheffield Teaching Hospital NHS Foundation Trust and University of Sheffield, Sheffield, United Kingdom; ^11^UCL Elizabeth Garrett Anderson Institute for Women’s Health, Medical School Building, University College London, London, United Kingdom; ^12^Cancer Clinical Trials Unit, University College London Hospitals NHS Foundation Trust, London, United Kingdom. ^13^Queen’s Department of Oncology School of Medicine, Queen’s Cancer Research Institute, Kingston, Canada; ^14^Italian Group for Adult Hematologic Diseases (GIMEMA), Health Outcomes Research Unit, Rome, Italy; ^15^Sydney Medical School, University of Sydney and Psycho-oncology Co-operative Research Group, School of Psychology, Faculty of Science, University of Sydney, Australia; ^16^NHMRC Clinical Trials Centre, University of Sydney, Sydney, Australia

###### **Correspondence:** Ameeta Retzer

**Abstract**

**Background & Aims**

Evidence suggests trial protocols often omit patient-reported outcome (PRO) content, potentially impairing PRO data collection, reporting, and subsequent impact. This qualitative study explored the factors influencing optimal PRO protocol content, implementation, and reporting, and the availability and use of PRO data during clinical interactions.

**Method**

Semi-structured interviews were conducted with four stakeholder groups: (1) Trialists and chief investigators of cancer clinical trials using a PRO as a primary or secondary outcome; (2) people with lived experience of cancer; (3) international experts in cancer clinical trial design and PROs; (4) journal editors, funding panellists, and regulatory board members. Data was analysed using thematic analysis with an iterative coding frame.

**Results**

Forty-four interviews were undertaken. Several factors affected whether PROs were effectively integrated into the trial and its findings. During the trial design, participants reported ambivalence towards PROs resulting in limited rationale and unclear means for their inclusion. Perceived lack of standardisation of PRO administration; concern relating to burden; and limited trial staff buy-in were seen to affect PRO data collection. Reporting was seen to be affected by the significance of the primary outcome or the PRO itself; the perceived ranking of PROs relative to the other outcomes by research teams; the perception that PRO findings were of lesser interest to journals; and restrictive word-counts. Strategies to address these were explored and many examples of good practice were identified.

**Conclusion**

The interviews indicated that misconceptions relating to PRO methodology and their use can undermine their planning, collection, and reporting. There is a role for regulatory, educational, methodological, and journalistic institutions to ensure that PRO training and guidance is available, signposted, and readily accessible to stakeholders, with accompanying measures to ensure adherence and compliance to best practice guidelines.

### 18) A New Patient-Reported Outcome Measure for Polymyalgia Rheumatica

#### Helen Twohig^1^, Sara Muller^1^, Caroline Mitchell^2^, Georgina Jones^3^, Christian Mallen^1^

##### ^1^Keele University, Keele, United Kingdom; ^2^University of Sheffield, Sheffield, United Kingdom; ^3^Leeds Beckett University, Leeds, United Kingdom

###### **Correspondence:** Helen Twohig

**Abstract**

**Background & Aims**

Polymyalgia rheumatica (PMR) causes pain, stiffness and associated disability in older adults. It usually has a sub-acute onset and responds rapidly to treatment with steroid medication, although the initial large improvement in health is typically followed by longer periods of lower level symptoms and episodes of relapse. Steroids themselves cause significant morbidity and adverse effects have to be balanced against PMR symptoms. Therefore, measuring the impact of PMR and its associated treatments from the patient’s perspective is of high importance. We have developed a patient-reported outcome measure (PROM) to assess PMR-related quality of life and present an overview of this process and our results from these studies.

**Methods**

**Scoping the problem:** systematic review of outcome measures used in studies of PMR, Patient and Public Involvement work

**Defining the construct:** qualitative study exploring 22 patient experiences of PMR

**Item development:** formation of items from the interview data, validation with participants.

**Pilot testing:** postal survey with a new group of 28 patients with PMR using the QQ-10 questionnaire to assess face validity, utility and feasibility.

**Item reduction and formation of dimension structure:** postal survey to gather responses from 256 people with PMR. Each individual completed the questionnaire twice, once according to how they felt now and once remembering back to how they felt at diagnosis. Classical and modern test theory methods used to refine the PROM and determine scoring.

**Results**

We have developed the first PROM evaluating PMR-related quality of life. It comprises two unidimensional scales fitting a Rasch model (a 9-item functional scale and a 4-item psychological well-being scale) as well as covering key symptoms and medication side effects.

**Conclusion**

We will evaluate the PROM’s validity, responsiveness and reliability in further studies to establish it as a tool fit for use in research and clinical practice.

### 19) Development of the content of the Sarcoma Assessment Measure (SAM)

#### Ana Martins^1^, Lindsey Bennister^2^, Lorna Fern^1^, Craig Gerrand^3^, Maria Onasanya^4^, Lesley Storey^5^, Mary Wells^6^, Jeremy Whelan^1^, Rachael Windsor^1^, Rachel Taylor^1^

##### ^1^University College London Hospitals NHS Foundation Trust, London, United Kingdom; ^2^Patient Representative, London, United Kingdom; ^3^The Royal National Orthopaedic Hospital, London, United Kingdom; ^4^Patient Representative, Manchester, United Kingdom; ^5^Queens University Belfast, Belfast, United Kingdom; ^6^Imperial College Healthcare NHS Trust, London, United Kingdom

###### **Correspondence:** Ana Martins

**Abstract**

**Background & Aims**

Introducing patient-reported outcome measures (PROMs) into clinical practice is known to improve patient-clinician communication, patient experience and outcomes. While there are many generic cancer PROMs there are none developed specifically for patients with sarcoma so these may not capture issues that are tumour-specific. This paper will report how the content of the Sarcoma Assessment Measure (SAM) was identified to reflect the issues patients with sarcoma face when living with and beyond diagnosis.

**Method**

The content of SAM has been developed systematically over a number of stages: 1. In-depth interviews were conducted with 121 patients: 50% male; aged 13-82; with STS (62%), bone (28%) and GIST (10%), 2. Content analysis of the interview transcripts identified 1,405 post-diagnosis experience statements 3. Experience statements were reviewed, repetition was removed and sentences were refined to form 395 ‘items’ which were included in an Item Reduction Questionnaire (IRQ) grouped as physical, emotional, social and financial wellbeing and sexuality. 4.The IRQ was completed by 250 patients: 51% male; aged 17-89; with STS (62%), bone (37%) and GIST (<1%), who rated each item on importance/worry. Items with a mean score above 5 (6 in the emotional domain), which reduced the list to 166 items. After review by researchers, clinicians and patients, 66 items were retained for the Content Validity Questionnaire (CVQ). 5. The CVQ was completed by 34 patients and 23 healthcare professionals. Items with a content validity ration of <.31 were removed. 6. Cognitive interviews were conducted with 10 patients on the final 22 items to test comprehension. Minor changes were made to four.

**Conclusion**

SAM comprises of 22 items reflecting physical, emotional, social, financial wellbeing and sexuality. This systematic process of using patient experience to develop the content of SAM will ensure it measures what is important to patients.

### 20) Exploring the variation of patient-reported outcome scores across different time-points (day, week, month) for patients with multiple conditions

#### Antoinette Davey^1^, Ian Porter^1^, Avril Mewse^2^, Colin Green^1^, Jose M Valderas^1^

##### ^1^University of Exeter Medical School, Exeter, United Kingdom; ^2^University of Exeter, Exeter, United Kingdom

###### **Correspondence:** Ian Porter

**Abstract**

**Background & Aims**

The effect of the timing of administration or completion of patient-reported outcome measurements (PROMs) has not been studied in detail, despite evidence of rhythmic fluctuations of symptoms people with chronic conditions experience (Smolensky et al 1999). Such fluctuations in symptoms can influence response to diagnostic tests and therapeutic interventions. An initial scoping review confirmed time-dependent variation in PRO scores across different chronic conditions, although there was a lack of qualitative research exploring explanations in the variations.

**Method**

This is a mixed-methods, longitudinal study spanning 9 months recruiting patients from primary care settings, diagnosed with 2 or more of the following conditions: asthma, depression and/or osteoarthritis. Participants were asked to complete paper/electronic generic and disease-specific PROMs a week prior to their interview. Interviews focused on their PRO scores, factors influencing their scoring, and what external (socially determined) and internal (mood, cognitive function) factors could impact on how they report their symptoms.

**Results**

A total of 17 patients with varying comorbidities of asthma, osteoarthritis and depression. Preliminary results indicated that fluctuations of PRO scores on disease-specific PROMs occur at different times of the day, with pain/stiffness for osteoarthritis patients at its worst in the morning and evening, asthma symptoms and depressive symptoms worse in the morning. This was influenced by external factors (e.g. what activities they were involved with, weather conditions) and recall of their health condition experience was affected by current health status, any recent attacks or hospitalisations, and mood. Additional analyses are being conducted on the interviews with full results to be presented at the conference.

**Conclusion**

The results pose potential questions regarding how timing of administration can affect scores and how variability of scores should be interpreted. Consideration needs to be given to the factors impacting on patient's appraisal process of their chronic condition(s) when completing PROMs.

### 21) Using Fourier analysis to examine variations in outcome scores for individuals with Meniere’s Disease

#### Antoinette Davey, Gary Abel, Colin Green, Jose M Valderas

##### University of Exeter Medical School, Exeter, United Kingdom

###### **Correspondence:** Antoinette Davey

**Abstract**

**Background & Aims**

Meniere’s disease is an incurable, chronic disorder of the inner ear, with patients experiencing varying levels of severity in hearing loss, tinnitus, aural fullness and vertigo, significantly impacting on patients’ quality of life, with increased incidences of social isolation amongst sufferers. There has been a lack of literature on how time of the day affects Meniere’s symptoms. Focusing on the fluctuating patterns of Meniere’s symptoms may support clinical practitioners in better understanding, supporting and diagnosing patients.

**Methods**

A pre-existing dataset was provided which used the Meniere’s Monitor mobile app to collect data from Meniere’s sufferers on a daily basis including their level of severity in dizziness, aura fullness, tinnitus, and hearing loss. Other questions regarding stress, sleep quality and demographics were collected. Data was collected between 2015 and 2017 and a total of 853 individuals provided data. Variability of symptom severity over a 24-hour period was assessed with a multivariate mixed-effects regression model using Fourier components. Time transformations, using sine and cosine functions, were created and added to the four main symptoms. Adjustments were made to differentiate trends from demographic factors.

**Results**

The majority of participants were female (68.3%), with a mean age of 48.9 years. Over half of the participants were employed (58.3%), and from Europe (54.2%). Peak aura fullness occurred between 4pm and 8pm compared to midnight. Tinnitus severity peaked at three points in the day - early morning (6am), midday (12pm), and 5pm. Dizziness symptoms peaked at different points in the day, mainly 10am and 3pm.

**Conclusion**

Usage of fourier transformation enabled variability of Meniere’s symptoms to be captured and analysed over a 24-hour period demonstrating peaks of symptoms at different times of the day. This analytic method would be useful for patients in better understanding and managing the disease ultimately affecting their overall wellbeing.

### 22) Ethnic group recruitment and utilization of appropriate patient reported outcomes in cancer clinical trials: current state of play

#### Anita Slade^1,2^, Ameeta Retzer^1^, Khalid Ahmed^3^, Derek Kyte^1,2^, Tom Keeley^4^, Jo Armes^5,6^, Julia Brown^7^, Lynn Calman^5,8^, Anna Gavin^5,9^, Adam Glaser^5,10^, Diana Greenfield^5,11^, Anne Lanceley^5,12^, Rachel Taylor^5,13^, Galina Velikova^10^, Grace Turner^1^, Melanie Calvert^1,2^

##### ^1^Centre for Patient Reported Outcomes Research, University of Birmingham, Birmingham, United Kingdom; ^2^NIHR Birmingham Biomedical Research Centre, University of Birmingham, Birmingham, United Kingdom; ^3^Birmingham Clinical Trials Unit, University of Birmingham, Birmingham, United Kingdom; ^4^Patient Centred Outcomes, GlaxoSmithKline, Uxbridge, United Kingdom; ^5^The National Cancer Research Institute POSCSG subgroup, London, United Kingdom; ^6^School of Health Sciences, University of Surrey, Guildford, United Kingdom; ^7^Clinical Trials Research Unit, University of Leeds, Leeds, United Kingdom; ^8^Macmillan Survivorship Research Group, Health Sciences, University of Southampton, Southampton, United Kingdom; ^9^N. Ireland Cancer Registry, Centre for Public Health, Queens University, Belfast, United Kingdom; ^10^Leeds Institute of Medical Research, St James’s University Hospital, Leeds, United Kingdom; ^11^Sheffield Teaching Hospital NHS Foundation Trust and University of Sheffield, Sheffield, United Kingdom; ^12^UCL Elizabeth Garrett Anderson Institute for Women’s Health, Medical School Building, University College London, London, United Kingdom; ^13^Cancer Clinical Trials Unit, University College London Hospitals NHS Foundation Trust, London, United Kingdom

###### **Correspondence:** Anita Slade

**Abstract**

**Background & Aims**

Patient reported outcomes (PROs) are increasingly used in cancer clinical trials to assess the impact of treatment on symptoms and quality of life. It is important that PRO data are collected from all appropriate cultural and ethnic groups within the target population in order to maximize the generalisability of the data. This study aimed to establish the extent to which different ethnic groups were represented in cancer trials collecting PROs, whether participants were included in PRO assessments, and the extent to which data were captured using culturally/linguistically validated measures.

**Methods**

National Institute for Health Research (NIHR) Portfolio Cancer clinical trials including PROs (2011-2014) were reviewed (n=228). We attempted to source matched trial protocols and publications to determine: (i) overall study sample ethnicity profiles; and (ii) whether PRO data were captured and reported using culturally/linguistically validated PRO measures. Semi-structured interviews with key stakeholders, explored the barriers and facilitators to recruitment and reporting of ethnic group PRO data in cancer trials.

**Results**

We identified 84 completed trials with matching protocols and publications. Only 14 (17%) of the included trials reported any ethnic profile data. Within these 14 studies, 611 (13%) of the total number of participants (n=4,754) were identified as belonging to non-white ethnic groups. None of the trial publications reported using culturally/linguistically validated PRO measures, despite the multi-national status of many of the trials.

Forty-four interviews were undertaken with international stakeholders including cancer trialists, regulators, policy makers and patient advocates. Participants discussed factors affecting optimal inclusion of ethnic group data, including recruitment challenges, limited community engagement by ethnic groups, resource limitations, and availability of appropriate translated PRO measures.

**Conclusion**

Greater transparency and increased efforts are required when reporting ethnic group data, and capturing important patient PRO data using culturally/linguistically validated measures in cancer clinical trials

### 23) A Systematic Review of Self-reported Quality of Life Measures for Adults with Mild to Moderate Learning Disabilities

#### John O’Dwyer^1^, Claire Hulme^2^, Louise Bryant^1^, Paul Kind^1^, David Meads^1^

##### ^1^University of Leeds, Leeds, United Kingdom; ^2^University of Exeter, Exeter, United Kingdom

###### **Correspondence:** John O’Dwyer

**Abstract**

**Background & Aims**

Around 1.2 million people in the UK have a mild or moderate learning disability, living on average 16 years less than the general population. Whilst there are clear health inequalities, research with this population is constrained. Evidence suggests this population often have difficulty completing research materials. This systematic review brings together evidence on self-reported quality of life (QoL) measures that have been used in research involving adults with a mild or moderate learning disability, and which have assessed their validity and reliability, to identify potential adaptations that might be made to the NICE recommended HRQoL measure, the EQ-5D, in order to reduce completion difficulty.

**Methods**

We included studies which contain self-reported QoL data of adults (>16 years) who have a mild or moderate learning disability. Searches were completed on Medline, EMBASE, Cochrane Library and other databases for English-language articles, reporting QOL data, published between 1990 and 2018. Screening and data extraction was completed by one reviewer and a random sample (10%) reviewed and validated by a second reviewer. A narrative synthesis was completed.

**Results**

From 22,291 unique citations identified, 218 studies formed the final dataset which included 47 self-reported QoL measures; 18 have been validated for use by adults with mild to moderate learning disabilities. These generic measures include various domains of QoL. Adaptations such as pictograms, contextualisation of language and longer completion times are reported.

**Conclusion**

The range of generic self-reported QoL measures used with this population is wide; comparison of measures is difficult as definitions of QoL, and hence domains examined, differ. The NICE reference case recommends using preference-based measures of HRQoL, with EQ-5D being their preferred measure in adults. Neither the EQ-5D nor any other preference-based measure has been validated for this population; Potential adaptations of EQ-5D to remedy this issue are discussed.

Prospero Registration: CRD42018092423 Funding: NIHR DRF 2017-10-159

### 24) The importance of pre-testing quality of life (QoL) items and measures: Two examples from a provisional QoL measure for primary sclerosing cholangitis (PSC)

#### Elena Marcus^1^, Bella Vivat^1^, Paddy Stone^1^, Douglas Thorburn^2^

##### ^1^University College London (UCL), London, United Kingdom; ^2^The Royal Free NHS Foundation Trust, London, United Kingdom

###### **Correspondence:** Elena Marcus

**Abstract**

**Background & Aims**

Primary sclerosing cholangitis (PSC) is a rare disease of the bile ducts and liver, which can impair quality of life (QoL). We are developing a new measure of QoL for people with PSC (PwPSC). The condition’s rarity means there is little relevant literature, so we identified potentially relevant issues from a survey with PwPSC, and QoL tools for co-morbid conditions or those with similar clinical features, e.g. inflammatory bowel disease (IBD).

**Method**

Following initial issue reduction, 83 retained issues were constructed as items for a provisional measure, and pre-tested with PwPSC in the UK. EM interviewed participants face-to-face or by telephone, exploring their understanding of items, and item acceptability, relevance, and redundancy.

**Results**

EM interviewed 24 PwPSC. Problems were identified with interpreting two items originating from QoL tools for IBD and colorectal cancer: (1) I have had frequent bowel movements; (2) I have had problems maintaining an ideal weight.

The bowel movement item (1) was interpreted both negatively (frequency being a burden) and positively (frequency perceived as healthy). People with co-morbid IBD tended to negative interpretations, whereas older people and/or those without IBD tended to positive interpretations.

The weight item (2) was always interpreted negatively, but participants understood “ideal weight” as requiring either weight loss or weight gain. Participants with mild symptoms, or those treated with steroids for IBD flare-ups, tended to be concerned about being overweight, whereas participants with more severe PSC were concerned about being underweight.

**Conclusion**

Our findings highlight the importance of pre-testing existing items in relevant target populations. PSC, as is common for rare conditions, presents heterogeneously, and so PwPSC may interpret items in distinct, sometimes opposing, ways depending on their particular health characteristics. Any potentially ambiguous items retained will be checked and re-phrased if necessary, to prevent future problems with item scoring.

### 25) “… for most of the times I was doing these forms I’m never dying”: what influences patients’ measured outcomes?

#### Linda Fenocchi, Gordon J. Hendry, Helen Mason

##### Glasgow Caledonian University, Glasgow, United Kingdom

###### **Correspondence:** Linda Fenocchi

**Abstract**

**Background & Aims**

Clinically important musculoskeletal (MSK) conditions of the lower leg, such as Achilles tendinopathy or plantar fasciitis, are prevalent and are frequently characterised by pain, loss of function and disability. PROMfoot was a prospective observational study to measure health outcomes following podiatric treatment for patients experiencing a new episode of foot pain. This included the completion of four patient reported outcome measures (PROMs), complemented by a qualitative study exploring the impact of foot pain on health-related quality of life (HRQoL) to inform, broaden and deepen understanding of underlying rationales behind responses to the PROMs.

**Methods**

A sub-sample of participants in PROMfoot took part in semi-structured interviews. Eligibility was completion of EQ-5D-5L, SF-12v2 (for SF-6D), Foot Function Index, and Foot Health Status Questionnaire at baseline and 3 months follow-up. Interviews were recorded digitally, transcribed verbatim, thematically coded and evaluated via a mixed methods approach with participant PROM scores during analysis.

**Results**

25 participants (f16:m9) were interviewed. 76% had self-reported at least one other comorbid condition. Generally, interviewees reported thinking more broadly than just the impact of their foot pain on HRQoL when approaching the completion of both EQ-5D-5L and SF-12v2. Themes were identified about severity of pain and variance of pain, adjustments to behaviour and activities, such as accommodating loss of function, and trade-offs between impacts of different health conditions.

**Conclusion**

Thematic analysis indicated that individuals consider different facets of health and health conditions for each question of EQ-5D-5L or SF-12v2. These could be unrelated to their foot pain. The influence of comorbid health conditions on interviewee’s judgement of their HRQoL was apparent in discussion. This highlights comorbidity and multi-morbidity as a potential confounder when measuring foot pain and podiatric treatment outcomes for economic evaluation.

### 26) Attitudes of care providers and patients towards an electronic patient reported outcomes (ePROs) to support care of patients with traumatic brain injury: a qualitative study (Priority)

#### Christel McMullan^1^, Ameeta Retzer^1^, Anita Slade^1,2^, Derek Kyte^1,2^, Laura Jones^1^, Antonio Belli^3^, Mel Calvert^1,2,3^, Grace Turner^1^

##### ^1^Centre for Patient Reported Outcome Research, University of Birmingham, Birmingham, United Kingdom; ^2^NIHR Biomedical Research Centre (BRC), Birmingham, United Kingdom; ^3^NIHR Surgical Reconstruction and Microbiology Research Centre (SRMRC), Queen Elizabeth Hospital, Birmingham, United Kingdom

###### **Correspondence:** Christel McMullan

**Abstract**

**Background & Aims**

Traumatic brain injury (TBI) is a leading cause of death and disability worldwide; over 50 million people have a TBI each year and global incidence is rising. Improvements in clinical management of TBI have resulted in improved survival rates; however, the consequence of this is more people living with life changing injuries and reduced quality of life. Electronic assessment of patient-reported outcomes (PROs) post-TBI may facilitate early identification of symptoms, facilitate shared-decision making and help improve long-term outcomes. This study aimed to: (i) establish the impact of TBI on patients’ quality of life; and (ii) to explore views on using ePROs to support clinical care and research.

**Methods**

Twenty-eight semi-structured one-to-one interviews were conducted with: (i) TBI survivors and family members/carers; (ii) healthcare professionals/researchers working in trauma related clinical areas; and (iii) members from third sector organisations supporting trauma patients and their families/carers. Data was analysed thematically.

**Results**

TBI led to significant impact on patients and families including cognitive, functioning, anxiety, and depression. All stakeholders were generally supportive of the development and use of an ePRO system as a flexible approach to identify, prioritise and evaluate ongoing symptoms and ensure that consultations focused on outcomes that matter to patients. However, a number of challenges were also identified including ensuring that patient symptoms are accurately reflected, difficulties in completion due to cognitive impairment or lack of insight. Key features of a new ePRO system include: simple layout, use of lay language, opportunity to send/receive feedback, and use of validated tools.

**Conclusion**

Positive attitudes towards ePROs demonstrate the potential to capture patient outcomes electronically in routine clinical practice and research. The next step is to co-design an e-PRO platform and test the usability, acceptability and feasibility and to inform system development in other areas of trauma research.

### 27) Developing an integrated national PROMs and PREMs platform for NHS Wales

#### Amanda Willacott

##### Cardiff and Vale University Health Board, Cardiff, United Kingdom

**Abstract**

**Background & Aims**

The all Wales PROMs, PREMs and Effectiveness Programme aims to collect PROMs and PREMs across secondary care. Its remit includes the development of an electronic portal to capture the data, integrated into the existing National Informatics Architecture.

**Methods**

The National Platform allows three electronic models of collection:

- A stand-alone e-form collected via tablets in clinic which requires clinic staff to load individual patients at each collection point. This system does not feed completed PROMs into the Electronic Patient Record.
- An admin portal that allows users to set up each patient for remote collection allowing patients demographics to be checked against the national Master Patient Index (MPI). This system allows completed PROMs to feed into the Electronic Patient Record.
- A remote system, integrated into the two main Patient Administration Systems used in NHS Wales secondary care, where patients PROMs/PREMs collection is automated where possible or administrative steps are aligned to existing workflows to reduce admin burden. This system feeds completed PROMs into the Electronic Patient Record.

**Results**

The National Platform is still in its development stages; however, implementation is on-going with collection of 29 of the 30 nationally agreed PROMs pathway via the system.

Over 25,000 PROMs have been collected to date with collection live across Wales, most of which are at point of referral into secondary care, however longitudinal collection is expanding with pockets of collection at post-treatment and follow up stages. Response rates range from near 100% with in-clinic collection, to 10% remotely at referral stage and 54% remotely post-surgery, based on one invite via letter.

**Conclusion**

However, these are indicative figures as more work is underway to integrate different models of communication (such as text and email reminders) to increase response rates. The team will share lessons learnt and demo the platform and plans for future improvements.

### 28) A taxonomy of short generic person-reported measures

#### Tim Benson

##### R-Outcomes Ltd, Thatcham, United Kingdom. UCL Institute of Health Informatics, London, United Kingdom

**Abstract**

**Background & Aims**

Commissioners and evaluators need to understand how health and care innovations help patients and staff, but lack tools needed to do this as part of routine care. For many innovations, the benefits cover multiple domains, but most patient-reported outcome and experience measures (PROMs and PREMs) address a single dimension and have been developed separately and in isolation.

**Method**

In collaboration with users, we have developed a family of short generic measures, for completion by patients, carers and staff. Each measure has a common look and feel, with four items and four response options each using emoji. Each item has a short heading, which is used in reporting, in addition to the wording of the question seen by respondents. Scores may be presented for individuals or for cohorts. Mean scores for cohorts are presented using a scale from 0 (all at floor) to 100 (all at ceiling).

**Results**

We have organised these measures as a taxonomy covering twenty-one short generic measures, which form a coherent family. The list covers: **Patient needs**: health status (howRu), personal wellbeing (PWS), health confidence (HCS), loneliness, sleep patterns and fatigue. **Treatment**: self-care, acceptance of loss, medication adherence and assessed need (howRthey). **Experience**: patient experence (howRwe), service integration, shared decision making (SDM). **Social factors**: social determinants of health (SDH), neighbour relations, staff relationships. **Innovations**: digital confidence, user satisfaction, innovation readiness, innovation process, behaviour change.

**Conclusion**

These measures have all been designed for digital data collection and results reporting using mobile devices. They can be used in combination on a pick and mix basis as part of short digital surveys. This taxonomy has proved useful in helping people understand the role of each measure, as well as to identify gaps and overlaps.

### 29) Evaluating the feasibility of electronically capturing patient-reported outcomes at the Royal Marsden Hospital

#### Emma Lidington, Helena Cho, Rachel Turner, Linda Wedlake, Clare Peckett, Eugenie Younger, Vicky LMN Soomers, Bernice Asare, Winette TA van der Graaf, Olga Husson

##### The Royal Marsden Hospital, London, United Kingdom

###### **Correspondence:** Emma Lidington

**Abstract**

**Background & Aims**

The Royal Marsden Hospital is evaluating the feasibility of implementing an electronic patient-reported outcome (ePRO) system (PROFILES). An interim analysis of outcomes important in assessing future use of the system was conducted in three currently recruiting studies.

**Methods**

Studies included the Young Adult Cancer Patient Journey (YACPJ), HOLISTIC and QUEST. YACPJ is a cross-sectional study recruiting patients of any malignancy, aged 25-39 by post. HOLISTIC and QUEST are prospective cohort studies with two-year follow-up at variable and fixed time points, respectively. In clinic, HOLISTIC recruit’s metastatic sarcoma patients undergoing chemotherapy. QUEST recruits recently-diagnosed sarcoma patients by post or in-clinic. Participants selected method of PRO completion. We assessed the proportion completing PRO questionnaires on paper vs. online, age (median and range), proportion participants that withdrew, and reasons for withdrawal. Age of respondents was compared using independent-samples t-tests.

**Results**

In YACPJ, 178(19.8%) patients participated of 901 invited, with 161(89.9%) completing online. No significant difference was found between participants completing online (median=37yrs) versus paper (median=38.5yrs; p=0.77). Twenty-three (62.6%) of 37 invited participated in HOLISTIC, with 18(78.3%) completing online. No significant age difference was found between online (median=61.5yrs) versus paper respondents (median=68.5yrs). Twelve (52.5%) patients withdrew: two formally withdrew; two ineligible; two lost to follow-up; three illness; three death. Forty-one (41.8%) of 98 invited participated in QUEST, with 21(51.2%) completing online. Online participants were significantly younger than paper (median=54v76yrs respectively; p<0.001). One patient withdrew due to illness.

**Conclusions**

Implementation of an ePRO system appears feasible across a wide range of ages (27–78yrs) and study designs. Whilst the majority of participants completed online, a sizeable number (43of200) completed on paper, suggesting that paper should remain an option. Higher response rates and uptake of online completion suggests future studies should favour in-clinic study invitation.

## Rapid report oral sessions

### 1) Are capability measures responsive to changes in vision? An exploration of the performance of the ICECAP-O in cataract surgery patients

#### Katie Breheny^1^, William Hollingworth^1^, Rebecca Kandiyali^1^, Padraig Dixon^1^, Mariusz Grzeda^1,2^, John Sparrow^1,2^

##### ^1^University of Bristol, Bristol, United Kingdom; ^2^Bristol Eye Hospital, Bristol, United Kingdom

###### **Correspondence:** Katie Breheny

**Abstract**

**Background**

The ICECAP-O is a preference-based measure (PBM) of capability-wellbeing in older-adults. To date, use of the ICECAP-O has not been reported in cataract patients. The relevance and responsiveness of preference-based health-related quality of life (HRQL) measures (e.g. EQ-5D) in visual disorders has been questioned. The benefits of cataract surgery might be better captured using broader measures than HRQL (such as capabilities), however the suitability of capabilities as an alternative has not been explored.

**Methods**

PREDICT-Cat is a UK cohort study of patients undergoing cataract surgery. Data was collected before surgery and 4-6 weeks after. All patients completed the ICECAP-O and Cat-PROM5, a validated measure of quality of life (QOL) impact of cataract. Patients were also randomised to complete either the EQ-5D-3L, EQ-5D-5L or EQ-5D-3L with vision bolt-on (EQ-5D-3L+VIS). The association between clinical outcomes (e.g. visual acuity), capabilities (ICECAP-O) and cataract QOL (Cat-PROM5) were explored and responsiveness to surgery evaluated.

**Results**

At baseline 1,308 patients completed the ICECAP-O, mean age 74.2. Minimal ceiling effects were observed at baseline for the ICECAP-O with 9.4% of patients scoring the maximum of one, compared to 8.7% (EQ-5D-3L+VIS), 27.1% (EQ-5D-3L) and 15.7% (EQ-5D-5L). ICECAP-O was moderately associated with cataract QOL (Spearman’s rho, -0.35) and patients with good vision had significantly better ICECAP-O scores. For responsiveness, effect sizes in patients reporting benefiting from surgery and improved visual QOL (anchors obtained from the post-operative Cat-PROM5) were 0.27 and 0.32 for the ICECAP-O, respectively. Effect sizes for other generic PBMs ranged from 0.13 (EQ-5D-5L, perceived benefit) to 0.20 (EQ-5D-3L, visual QOL improvement).

**Conclusions**

PREDICT-CAT provides a valuable resource to examine the performance of PBMs in vision, demonstrating that ICECAP-O has a low ceiling effect and is responsive to patient-reported benefits. Results suggest that capability measures could be an alternative to HRQL outcomes when assessing the cost-effectiveness of cataract surgery.

### 2) Use of web-based surveys for the administration of patient-reported outcome measures (PROMs)

#### Charlotte Panter, Helena Bradley, Katie Tinsley

##### Adelphi Values Ltd, Bollington, United Kingdom

###### **Correspondence:** Helena Bradley

**Abstract**

**Background & Aims**

Web-based surveys are a popular alternative to traditional forms of data collection (e.g., pen-and-paper surveys or interviews) in health outcomes research. Patient Reported Outcome Measures (PROMs) provide valuable insights into the experience of health conditions directly from the patient’s perspective. The challenges, considerations and solutions associated with the use of web-based surveys for the administration of PROMs are presented, informed by multiple real-life examples.

**Method**

Web-based surveys provide an efficient, convenient and cost-effective method for collecting data. Identification and enrolment of respondents via online platforms (e.g. survey links shared on patient advocacy websites and social media pages) facilitates the collection of data from geographically diverse samples within short timeframes. Incorporating validated PROMs into a web-based surveys can provide valuable insights into patient’s disease and treatment experiences. Administration of PROMs via web-based systems can reduce researcher bias (e.g., minimising social desirability bias) and improve scientific rigour (e.g., avoidance of secondary data errors).

**Discussion**

Implementation of PROMs within web-based surveys should be informed by the research objectives, study design and target population. Key considerations include: target population (e.g. eligibility criteria, achieving a representative sample, online presence/activity); cross-sectional versus longitudinal designs (accounting for challenges of keeping patients engaged over long periods of time); and PROM selection (e.g. relevance, completion time, available formats). Obtaining a clinician-confirmed diagnosis of the condition may be challenging and the risk of completions by ineligible participants (especially where honoraria is included) should be considered. Strategies can be employed to reduce the impact of these challenges, including ways of confirming respondent eligibility, facilitating recruitment and meeting sampling quotas, mitigating inattentive or ineligible completions and overcoming “time-outs” (participant withdrawal).

**Conclusion**

With careful planning and acknowledgement of potential challenges, web-based systems are an efficient, convenient and cost-effective method for collecting real-world PROM data directly from patients.

### 3) electronic Patient self-Reported outcomes to Improve cancer Management and patient Experiences (ePRIME) – a Delphi consultation informing the development of a community-based follow-up intervention for ovarian cancer patients

#### Leanne Shearsmith^1^, Kennedy Fiona^1^, Chris Bradley^2^, Uschi Hoffman^3^, David Jackson^4^, Dan Lee^5^, Geoff Hall^4^, Julia Brown^6^, Galina Velikova^1^

##### ^1^Unversity of Leeds, Leeds, United Kingdom; ^2^Bradford Teaching Hospital NHS Foundation Trust, Bradford, United Kingdom; ^3^Calderdale & Huddersfield NHS Foundation Trust, Huddersfield, United Kingdom; ^4^Leeds Teaching Hospitals NHS Trust, Leeds, United Kingdom; ^5^Airedale NHS Foundation Trust, Keighley, United Kingdom; ^6^Clinical Trials Research Unit, University of Leeds, Leeds, United Kingdom

###### **Correspondence:** Leanne Shearsmith

**Abstract**

**Background & Aims**

Improvements in cancer treatment have led to many patients being cured or in remission, but requiring follow-up to detect recurrence, and manage symptoms/side effects. With growing numbers of patients, traditional hospital-based follow-up is not sustainable. New models of follow-up care and long-term symptom tracking are needed and electronic patient-reported outcome measures (ePROMs) may facilitate these new pathways.

**Aims**

To inform the development of the ePRIME follow-up intervention for ovarian cancer. Routinely these patients are seen face-to-face 3-monthly and CA125 monitored. ePRIME aimed to explore the potential use of ePROMs, CA125 monitoring, plus a nurse-led telephone consultation instead of routine clinic visits.

**Methods**

Across 4 hospitals, clinicians and patients (6 months-3 years post-treatment) were invited to participate in a Delphi consultation to identify the key symptoms to monitor post-treatment, the PROMs items to measure these and the frequency.

**Results**

17 patients and 12 staff took part in round 1, and 11 patients and 8 staff round 2.  Key symptoms included: abdominal pain/discomfort, bloating/swelling, nausea/vomiting, appetite loss, diarrhoea/constipation, urinary symptoms, shortness of breath, fatigue, swollen legs and unexpected weight change. Most agreed on patients being asked about the symptom’s duration, frequency and whether it has changed recently. Psychological wellbeing/holistic needs were also viewed as important.  In round 2 more staff and patients favoured the format of the patient-worded toxicity items and felt that completing 3-monthly was reasonable but with access earlier if required.

**Conclusions**

The ePRIME follow-up intervention is currently being evaluated in a small pilot study.

### 4) Symptom Burden After Living Donor Kidney Transplant Measured with the Functional Assessment Of Cancer Therapy - Kidney Symptom Index Is Associated With Worse Transplant Outcomes

#### Devin Peipert^1,2^, Juan Caicedo^2^, Kelse Ensor^2^, John Friedewald^2^, Michael Abecassis^2^, David Cella^1,2^, Daniela Ladner^2,1^, Zeeshan Butt^1,2^

##### ^1^Department of Medical Social Sciences, Northwestern University Feinberg School of Medicine, Chicago, USA; ^2^Northwestern University Transplant Outcomes Research Collaborative, Comprehensive Transplant Center, Feinberg School of Medicine, Chicago, USA

###### **Correspondence:** Devin Peipert

**Abstract**

**Background & Aims**

Regulators and other stakeholders are seeking valid patient reported outcome measures (PROMs) to evaluate treatments for kidney transplantation (KT). To examine the validity of a PROM-based symptom assessment for living donor KT (LDKT) recipients, we determined frequency and severity of symptoms on the Functional Assessment of Cancer Therapy - Kidney Symptom Index (FKSI-19) and associations with health-related quality of life (HRQOL) and failure of the transplanted kidney graft.

**Method**

We assessed symptoms at 3 mo and 1-year post-LDKT among 404 recipients between 11/2007 and 08/2016 using the FKSI-19. The FKSI-19 includes 19 symptoms rated from “not at all” to “very much”. We examined associations between FKSI-19 overall scores, as well as individual symptoms, with 1-year post-LDKT outcomes, including: 1) HRQOL; 2) death censored graft survival (DCGS)

**Results**

The symptoms most commonly rated as severe were: sleeping difficulties (21% at 3 mo. and 1 year) and fatigue (17% at 3 mo., 13% at 1 year). Patients with severe fatigue had lower scores on the SF-12 Physical Component Summary: 38.8 vs. 50.2 (p<0.001; Cohen’s d = -1.25) at 1-year post-LDKT. Similarly, patients with worse appetite (49.9 vs. 54.2; p<0.001; Cohen’s d = -0.53) and severe sleeping difficulties (49.2 vs. 54.8; p<0.001; Cohen’s d = -0.63) scored lower on the on the SF-12 Mental Component Summary. Patients with lower than median FKSI-19 scores at 3 mo. post-LDKT had significantly lower 1 -year DCGS (94.5% vs. 98.4%, log rank p=0.04). In addition, specific symptoms at this timepoint were associated with lower DCGS, including fatigue (91.8% vs. 97.4%, log rank p=0.03) and sleeping difficulties (90.9% vs. 97.4%, log rank p=0.02).

**Conclusion**

Symptom severity after LDKT as assessed on the FKSI-19 is an indicator of risk for graft loss. The FKSI-19 may be leveraged as a measure for evaluating post-LDKT health and treatment.

### 5) Optimising Patient and Public Involvement in Patient Reported Outcome Measure (PROM) development

#### Jill Carlton^1^, Tessa Peasgood^1^, Samaira Khan^1^, Rosemary Barber^1^, Jennifer Bostock^2,3,4^, Anju Devianee Keetharuth^1^

##### ^1^Univeristy of Sheffield, Sheffield, United Kingdom; ^2^University of Cambridge, Cambridge, United Kingdom; ^3^University of Oxford, Oxford, United Kingdom; ^4^King’s College London, London, United Kingdom

###### **Correspondence:** Jill Carlton

**Abstract**

**Background & Aims**

Public involvement (PI) in PROMs selection and development is a priority. However, there are potential difficulties in identifying where PI can occur in the different stages. We aimed to propose a framework to identify where PI *could* occur at each stage of PROM development. The stages described identify potential activities that can be undertaken when developing or refining a PROM.

**Methods**

Eleven stages of PROM development were identified, and within each of these PI activities may be undertaken. These include: 1) establishing a need for a new or refined PROM; 2) devising a conceptual model; 3) identifying item content; 4) item development; 5) item reduction; 6) pre-testing of items (cognitive interviews and debriefing); 7) psychometric survey design; 8) psychometric survey analysis; 9) selection of items for the PROM; 10) design of the PROM; 11) dissemination and promotion of the PROM. PI activities may include reviewing and critiquing existing evidence; input on study design, culturally appropriate issues, participant-facing documents, ethical considerations, input and advice on interpretation of results, advice on format and layout of PROM, advice on strategies for wider dissemination, and co-authorship and co-presenting.

**Results**

This emerging framework sets out ways in which PI can have a meaningful role and contribution to the co-development of PROMs. Incorporating PI is an important part of this process, and its inclusion contributes to strengthening the relevance, acceptability and validity of the PROM itself. This framework is not prescriptive as the sequence of PROM development is not uniform. The type and level of PI will vary between studies.

**Conclusion**

There have been calls for clarity, guidance and consensus on PI in PROM development. This emerging framework is a response to those requests and a contribution to the ongoing dialogue on PI in PROM development.

### 6) The development of a new patient reported experience questionnaire for the assessment of the bladder cancer treatment pathway

#### Alan Uren, Nikki Cotterill, Paul Abrams, Edward Rowe

##### Bristol Urological Institute, Bristol, United Kingdom

###### **Correspondence:** Alan Uren

**Abstract**

**Background & Aims**

There are more than 10,000 bladder cancer diagnoses in the UK every year. Bladder removal (cystectomy) is the standard treatment with an ileal conduit (with external stoma bag). The complex patient pathway can result in unmet patient needs during diagnosis, treatment and recovery. In particular, there is a drive nationally to improve the recording of surgical outcomes (e.g. post-operative complications). Most importantly, Patient Reported Experience Measures (PREMs) allow the objective measurement and assessment of a particular service or aspect of patient care from the patient perspective. However, existing PREMs are often lengthy and applicable to the general cancer pathway or other specific cancer types.

**Objective:** The development of a fully validated PREM that allows comparison across cancer centres, to drive measurable improvements to bladder cancer patient care quality.

**Method**

Stage 1: Semi-structured interviews with fourteen patients who had received a cystectomy over the last 18 months were used to explore the patient experience of the bladder cancer pathway. Thematic analysis of the transcripts was used to categorise the experiences of the patients. An expert clinical panel were also consulted for their opinion on a hypothesised inventory of core items, evidenced from the literature review and initial interviews.

**Results**

Our interviews highlighted the importance to patients of a timely referral, a sensitive explanation of the diagnostic results and risk/benefits of treatment, and the impact of surgical complications.

**Conclusions and prospective development**

Stage 2: Cognitive interviews with patients are underway to assess respondent understanding of a draft instrument, including the facility of its completion, and comprehension of the items and response scales. Stage 3: The resulting draft questionnaire will then be pilot tested in the patient population to inform further fmodifications and item number reduction.

### 7) Self-completion and proxy measurements for quality of life in children? Lessons from a pilot study on children with behavioural problems.

#### Sarah Abraham^1^, Sandy Tubeuf^2^

##### ^1^University of Leeds, Leeds, United Kingdom; ^2^Université Catholique de Louvain, Louvain-la-Neuve, Belgium

###### **Correspondence:** Sarah Abraham

**Abstract**

**Background & Aims**

There is a debate in the health outcomes literature regarding who the most appropriate respondent is when assessing children’s health-related quality of life (HRQoL). In some cases, parent-proxy may be the only practical option where children are unable to self-complete a HRQoL questionnaire. However, children’s self-reported values may be preferable because HRQoL is subjective and represents one's own perception of health. We collected EQ-5D-3L Youth version (EQ-5D-Y) as part of a feasibility study comparing child psychotherapy with usual care for children with conduct disorders aged 5-11 years. The questionnaire was self-completed at baseline and 4 months follow-up by the child via face-to-face researcher administration when possible and by one parent as a proxy respondent.

**Method**

We presented percentage of completion for each questionnaire at each time point. We performed descriptive analysis to see if missing data were related to child age. We also investigated level of agreement between each of the 5 dimensions and the visual analogue scale of EQ-5D-Y when self-completed by the child or by proxy-respondent.

**Results**

A total of 32 dyads (16 in each arm) participated in the study. About two thirds of children (65.5%) were able to complete the EQ-5D-Y at baseline, and 34.4% at follow-up. There was no proxy-respondent missing data at baseline while 25% did not complete at follow-up. Age appeared unrelated to child completion. Children and primary carers were concordant regarding the child’s health. The visual analogue scale was also concordant with children responses (78 baseline, 80.9 at 4 months) and proxy-respondent responses (75.6 baseline, 79.17 at 4 months).

**Conclusion**

The assessment of quality of life by children using self-report questionnaires was possible with the help of a face-to-face researcher. Parents appeared as an appropriate second best when children are unable to self-complete the EQ-5L-Y.

